# Attention and feature transfer based knowledge distillation

**DOI:** 10.1038/s41598-023-43986-y

**Published:** 2023-10-26

**Authors:** Guoliang Yang, Shuaiying Yu, Yangyang Sheng, Hao Yang

**Affiliations:** School of Electrical Engineering and Automation, Jiangxi University of Science and Technology, Ganzhou, 341000 Jiangxi China

**Keywords:** Computer science, Information technology

## Abstract

Existing knowledge distillation (KD) methods are mainly based on features, logic, or attention, where features and logic represent the results of reasoning at different stages of a convolutional neural network, and attention maps symbolize the reasoning process. Because of the continuity of the two in time, transferring only one of them to the student network will lead to unsatisfactory results. We study the knowledge transfer between the teacher-student network to different degrees, revealing the importance of simultaneously transferring knowledge related to the reasoning process and reasoning results to the student network, providing a new perspective for the study of KD. On this basis, we proposed the knowledge distillation method based on attention and feature transfer (AFT-KD). First, we use transformation structures to transform intermediate features into attentional and feature block (AFB) that contain both inference process information and inference outcome information, and force students to learn the knowledge in AFBs. To save computation in the learning process, we use block operations to align the teacher-student network. In addition, in order to balance the attenuation ratio between different losses, we design an adaptive loss function based on the loss optimization rate. Experiments have shown that AFT-KD achieves state-of-the-art performance in multiple benchmark tests.

## Introduction

The success of convolutional neural networks (CNN) has brought profound changes to the field of computer vision^1^. However, in order to achieve performance improvements, CNN continue to scale, and with them, compute and storage requirements increase, which is a huge challenge for deployment on edge devices with limited resources. In order to obtain efficient and compact networks, model compression methods such as low-rank decomposition^2–6^, network pruning^7–11^, quantization^12–14^, efficient network architecture design^15–17^, and distillation^18–20^ have been rapidly developed. Among them, knowledge distillation (KD) is a promising method.

KD forces the student network to extract knowledge from the larger teacher network in order to improve performance. KD, first proposed in^21^, is a representation of logic-based knowledge distillation. In recent years, feature-based and attention-based KD methods have emerged. Feature-based KD enables the student network to extract knowledge from the middle and deep features of the teacher network, which is characterized by the high precision of the student network. In the logic-based KD approach, knowledge is extracted from the logical output of the teacher network, and the student network is supervised by both the truth label and the teacher logic. The attention-based KD method is proposed in^30^, which forces students to network simulate the attention map transferred from the teacher in order to learn what valuable content should be paid attention to in the classification process.

**Figure 1:**
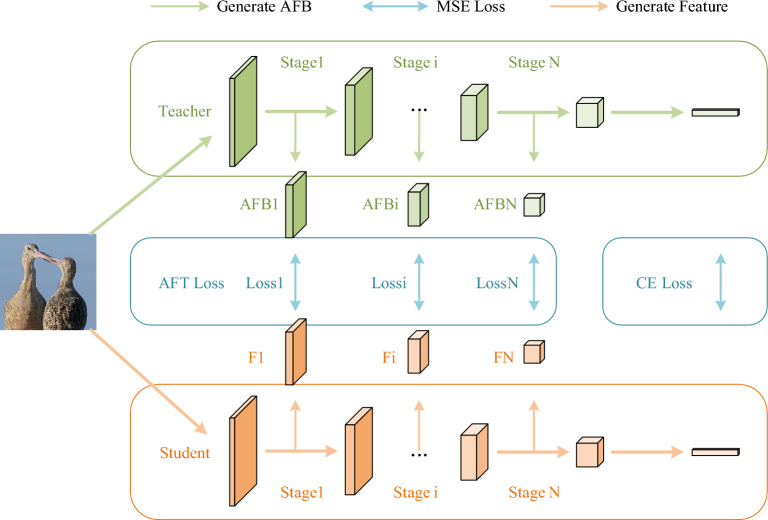
Illustration of AFT-KD. To align the teacher and student network, we divide it into N stages of reasoning. The value of N varies in different teacher-student structures. For example, N equals 3 in the experiment in section "Exploration of AFB".

The existing methods divide knowledge distillation into three types according to the types of knowledge transfer: feature based, logic based and attention diagram, and focus on how to use a single feature to improve the performance of the network. Unlike existing methods, our goal is to investigate how more comprehensive inference information can be used to improve the performance of distillation. Therefore, we re-examine the CNN architecture and find that the convolutional layer in CNN is not only spatially continuous, but also shows time continuity in the forward transmission of data. In the continuous inference process from input feature to output category prediction, the feature map and the logical prediction represent the inference results of the intermediate inference stage and the whole inference process respectively, while the attention map reflects what the convolution layer pays most attention to in the inference process, which contains the knowledge related to the inference process. Based on the above findings, we reclassify the existing KD methods into two types: inference process based and inference result based, and the distillation methods based on feature and logic in the traditional classification are collectively called inference result based distillation methods. When we examine the KD method from this new perspective, we find that the existing methods only use one of the inference process information or the inference result information, and ignore the correlation between the two. In response to this phenomenon, we propose a knowledge distillation method based on attention and feature transfer (AFT-KD, Fig. 1), which transfers the intermediate features and their corresponding attention attempts to the student network at the same time to achieve better performance..

**Figure 2:**
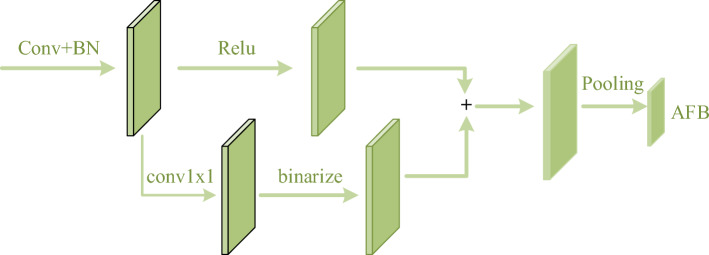
Illustration of AFB generation method.

Our work mainly consists of two parts. The first part is about how to get inference information. First, we find the extensibility of^19^, where the operation of generating a class activation map (CAM) by projecting the weights of the output layer back into the convolutional feature map can be easily extended to other convolutional layers. Inspired by^30^, we use 1 × 1 point convolution to generate the attention map corresponding to the intermediate feature map and superimpose it on the original feature map after binarization (Fig. 2), which we call the attention and feature block (AFB). Then, according to the structure of CNN, we divided it into different stages to simulate different reasoning moments, and forced the student network to approach the AFB of all stages. The advantage of this is that students can learn the complete reasoning process, and the block operation reduces the required calculation amount. The second part is about how to balance the loss function. We refer to the error generated by approximating AFB as KD Loss and the error between the predicted output and the truth label as cross entropy Loss (CE Loss). In order to balance the rate of optimization between KD Loss and CE Loss and prevent the loss of accuracy due to continued training after convergence of one loss, we designed an Adaptive loss function to adjust the loss weight using the ratio of the loss decay rate to the expected rate of the two loss decay rates.

Overall, our contributions are summarized as follows:

- We reclassify the existing KD methods into inference process based and inference result based, reveal the relationship between the existing KD methods, and provide a new perspective for the research of knowledge distillation.
- We design an efficient strategy to balance the optimal rate between distillation losses and cross-entropy losses, and it is easy to scale to multi-task learning scenarios.
- We propose a knowledge distillation method based on attention and feature transfer, named AFT-KD, which achieves state-of-the-art performance across multiple benchmarks.

Finally, we use a total of five chapters to arrange the content of the article. The second chapter introduces the related work of various knowledge distillation methods according to the traditional classification method, and analyzes the connection and deficiency between different methods. The third chapter introduces the proposed method in detail, including CAM review, AFT-KD theoretical analysis and Adaptive Loss implementation. The fourth chapter contains all the experimental content. First, we introduced the data set used in the experiment, then analyzed the influence of the information contained in AFB on the distillation performance, and further verified the performance superiority of AFB-learned AFB method. Finally, we verified and analyzed the actual performance of Adaptive Loss. In the last chapter, we summarize the proposed methods and analyze their limitations and what we will do next.

## Related work

The concept of knowledge distillation (KD) was proposed by Hinton et al.^21^, which forced the student network to extract knowledge from the soft labels and ground truth labels provided by teachers. In order to make full use of the "dark knowledge" contained in soft labels, the concept of temperature was introduced. The existing KD methods can be mainly divided into three types: logic-based^20,21,31–34^, feature-based^18,22–29,35^, and attention maps-based^19,30^.

Logic distillation transfers the knowledge implicit in the output logic of the teacher model to the student network. BAN^32^ obtained superior performance to the teacher model by directing the same parameterized network as the teacher. DKD^20^ reformulates KD loss into target-class knowledge distillation (TCKD) and non-target-class knowledge distillation (NCKD), revealing that KD's coupling formula limits the effectiveness and flexibility of knowledge transfer. CrossKD^34^ passes intermediate features of the student network to the teacher's detection head, resulting in cross predictions, which are then forced to mimic the teacher's predictions. In addition, there are several articles on logical distillation methods^21,33,34^.

Feature-based KD methods tend to have better performance, forcing students to extract valid content from intermediate features of the teacher network at the cost of requiring more computation than logical distillation. RKD^25^ can transform the relationship of data examples to punish differences in teacher and student relevance, similar to the transfer of sample relevance studies from teacher and student networks^26,27^. PKT^35^ models the teacher's knowledge as a probability distribution and uses KL divergence to measure distance. RKD^25^ uses multi-case relationships to guide students' learning. CRD^22^ combines comparative learning with knowledge distillation, and uses comparative objectives to carry out knowledge transfer. ReviewKD^18^ uses cross-layer connection paths to integrate the knowledge implied by features at different levels.

KD method based on attention diagram instructs students what information the network should pay attention to in reasoning. AT^30^ verifies the validity of shifting attention diagram, which uses class activation graph to transfer knowledge to student network. CAT-KD^19^ reveals that the ability to distinguish category regions is the key to network classification, and proves that this ability can be acquired and enhanced by transferring CAM. CAT-KD can transfer knowledge by transforming structure to obtain attention force, which makes attention-based knowledge distillation has a good competitiveness.

The KD method based on transfer logic and features has good performance, while the KD method based on transfer attention diagram has high interpretability. Previous studies have ignored the link between these two characteristics. In this paper, we aim to solve this problem by proposing a KD approach based on attention and feature transfer, which is advanced in several benchmark tests.

## Our method

In this section, we first review CAM and analyze its scalability, then further propose the attention-feature fusion AFB and apply it to knowledge distillation, and finally propose the adaptive Loss function combined with the decay rate of KD Loss and CE Loss.

### Review the CAM

First we consider A commonly used CNN structure with the output feature $A \in {\mathbb{R}}^{C \times H \times W}$ of its last convolutional layer, where C represents the number of channels and H and W represent the height and width of the feature, respectively. $A_{i} \in {\mathbb{R}}^{H \times W}$ denotes the feature of the i-th channel.$A_{i} \left( {x,y} \right)$ denotes the activation at spatial location $\left( {x,y} \right)$ on channel i. At this point, the process of generating prediction results by regular CNN can be expressed as follows.

1 $$\begin{aligned} P_{j} & = \sum\limits_{1 \le i \le C} {W_{i}^{j} GAP\left( {A_{i} } \right)} \\ & = \frac{1}{W \times H}\sum\limits_{x,y} {\sum\limits_{1 \le i \le C} {W_{i}^{j} A_{i} \left( {x,y} \right)} } \\ \end{aligned}$$

where $P_{j}$ represents the logical prediction of the j-th category, and $W_{i}^{j}$ represents the weight corresponding to the j-th category in the fully connected layer. In^30^, the class activation graph (CAM) is generated by projecting the weight of the fully connected layer in CNN back to the convolutional feature map, so we can get the calculation formula corresponding to CAM for the j-th category:

2 $$CAM_{j} = \sum\limits_{1 \le i \le C} {W_{i}^{j} A_{i} }$$

Equation (2)can also be rewritten as:

3 $$CAM_{j} \left( {x,y} \right) = \sum\limits_{1 \le i \le C} {W_{i}^{j} A_{i} } \left( {x,y} \right)$$

According to Eqs. (1 and 3), the relationship between logical output $P_{j}$ and logical output $CAM_{j}$ is as follows:

4 $$\begin{aligned} P_{j} & = \frac{1}{W \times H}\sum\limits_{x,y} {CAM_{j} \left( {x,y} \right)} \\ & = GAP\left( {CAM_{j} \left( {x,y} \right)} \right) \\ \end{aligned}$$

As shown in Eq. (4), the logical output of class j can be obtained by calculating the average of the corresponding class CAM. This kind of operation to obtain further inference results through feature mapping is very common in CNN. For example, the feature graph generated by the convolution kernel is regularized and activated to obtain the final conclusion. Therefore, we can easily generalize the CAM calculation process to other locations in the CNN, and the generated CAM-like map corresponds to the output feature map of the layer.

### AFT-KD

To gain knowledge related to the inference process, we extend CAM to all convolutional layers of the CNN. First, we formulate the general operation of the convolution layer, assuming that CNN contains L convolution layers, where the input feature of the k-th convolution layer is $F_{k - 1} \in {\mathbb{R}}^{C \times H \times W}$, and C, H, W represents the channel number, height and width of the input feature respectively. Then the input feature of the k + 1 convolution layer is:

5 $$\begin{aligned} F_{k} & = ACT_{k} \left( {Norm_{k} \left( {Conv_{k} \left( {F_{k - 1} } \right)} \right)} \right) \\ & = ACT_{k} \left( {\Pr e\_F_{k} } \right) \\ \end{aligned}$$

where $ACT_{k} \left( {\text{g}} \right),Norm_{k} \left( {\text{g}} \right),Conv_{k} \left( {\text{g}} \right)$ represents activation function, regularization function and convolution operation of the k layer respectively. $\Pr e\_F_{k}$ represents the pre-activated output feature. We approximate the activation process of the pre-activated output feature as the mapping from the inference process to the inference result. In order to extract knowledge about the inference process in $F_{k}$, we use the pre-activated output feature $\Pr e\_F_{k}$ to compute the class CAM corresponding to $F_{k}$. Inspired by^18^, we use point convolution to replace the weight of the fully connected layer, then formula 3 can be rewritten as:

6 $$CAM_{j} = conv_{j} \left( A \right)$$

where $conv_{j} \left( {\text{g}} \right)$ represents the convolution kernel of the j-th channel corresponding to the output feature. Combined with Eq. (6), the attention map corresponding to $F_{k}$ can be expressed as:

7 $$CAM_{k}^{L} = Bin\left( {conv_{k} \left( {\Pr e\_F_{k} } \right)} \right)$$

where $CAM_{k}^{L}$ represents the CAM class corresponding to $F_{k}$. $Bin\left( {\text{g}} \right)$ stands for binarization, and using binarization operations can further highlight what convolution is concerned with when reasoning. Finally, we superposition $CAM_{k}^{L}$ and $F_{k}$ to obtain the output feature that integrates the inference process and inference result, which is called Attention and Feature Block (AFB). The calculation process of attention and feature block of the k-th convolutional layer is as follows:

8 $$AFB_{k} = CAM_{k}^{L} + F_{k}$$

On this basis, we propose AFT-KD, which forces students to simulate the knowledge in AFB transferred by teachers. In order to save computation and align the student and teacher networks, we divide CNN into N inference stages, where AFT losses can be defined as:

9 $$\begin{aligned} L_{AFT} & = \sum\limits_{1 \le k \le N} {L_{AFT}^{k} } \\ & = \sum\limits_{1 \le k \le N} {\sum\limits_{{1 \le j \le C_{k} }} {\frac{1}{{C_{k} }}} } \left\| {\frac{{\phi \left( {AFB_{k}^{j} } \right)}}{{\left\| {\phi \left( {AFB_{k}^{j} } \right)} \right\|_{2} }} - \frac{{\phi \left( {F_{k}^{j} } \right)}}{{\left\| {\phi \left( {F_{k}^{j} } \right)} \right\|_{2} }}} \right\|_{2}^{2} \\ \end{aligned}$$

where, $C_{k}$ represents the number of channels in the output feature graph of the k stage after adjustment. $\phi \left( {\text{g}} \right)$ is the adjustment function used to unify the number of channels and resolution of $AFB_{k}$ and $F_{k}$. $F_{k}$ is the output feature map of the k stage student network.

### Adaptive loss

In order to balance the optimization rate between different losses, we design an adaptive loss function based on the loss optimization rate. After determining AFT losses, we define the overall losses of AFT-KD as:

10 $$L_{KD} = \alpha L_{CE} + \beta L_{AFT}$$

where $L_{CE}$ represents the standard cross entropy loss, $\alpha ,\beta$ is the dynamic hyperparameter used to balance $L_{CE}$ and $L_{AFT}$. When the optimization rates of $L_{CE}$ and $L_{AFT}$ are different, there will be a phenomenon of premature convergence of a certain loss in training. In this case, continuing to train the network can reduce the unconvergent loss function, but at the cost of sacrificing the precision of the convergent task. In order to avoid this phenomenon, we introduce the loss optimization rate to balance the decay rate of the two loss functions.

First, we need to record the initial loss $L_{CE}^{0}$ and $L_{AFT}^{0}$ at the beginning of training, and record the current loss $L_{CE}$ and $L_{AFT}$ at any iteration. At this point we can calculate the loss attenuation rate in this iteration:

11 $$\left\{ {\begin{array}{*{20}c} {Dr\_CE = L_{CE} /L_{CE}^{0} } \\ {Dr\_AFT = L_{AFT} /L_{AFT}^{0} } \\ \end{array} } \right.$$

where $Dr\_CE$ and $Dr\_AFT$ are respectively the attenuation rates of $L_{CE}$ and $L_{AFT}$ relative to the initial loss in this round of iteration. The average attenuation rate $\overline{D}r = (Dr\_CE + Dr\_AFT)/2$, $\alpha ,\beta$ can be expressed as:

12 $$\left\{ {\begin{array}{*{20}c} {\alpha = Dr\_CE/\overline{D}r} \\ {\beta = Dr\_AFT/\overline{D}r} \\ \end{array} } \right.$$

According to Eqs. (11 and 12), for the task that is optimized faster, its dynamic coefficient is a positive number less than 1 in this round, and the faster the optimization, the smaller the coefficient, which will reduce its optimization efficiency in this round to achieve the purpose of balancing another task. The experiment shows that the adaptive loss can effectively shorten the distance between $Dr\_CE$ and $Dr\_AFT$.

## Experiments

### Datasets and implementation details

#### Datasets

Our experiments are mainly conducted on two image classification datasets:

1. CIFAR-100^36^ contains a total of 60,000 32 × 32 pixel pictures in 100 categories, among which the training set and the verification set contain 50,000 and 10,000 pictures respectively.
2. ImageNet^37^ is a large-scale dataset containing 1000 classification objects, including 1.2 million training images and 50,000 verification images.

#### Implementation details

Our experimental setup on CIFAR100 and ImageNet is strictly followed^19,20^. In the lab on CIFAR100, we trained 240 epochs using the SGD optimizer with the batch size set to 64. The initial learning rate of 0.05 (0.01 for ShuffleNet^38,39^ and MobileNet^17^) was divided by 10 at 150, 180, and 210 iterations. For the experiment on ImageNet, we trained 100 epochs with a batch size of 512. The initial learning rate is 0.2, and every 30 epochs decays to one-tenth of the original. In addition, we conducted experiments on various representative CNN networks :VGG^40^, ResNet^41^, WideResNet^42^, MobileNet^17^, and ShuffleNet^38,39^. Table 1 provides a brief overview of these networks.

**Table 1:** Several common neural networks are briefly introduced.

VGG^40^	VGG is constructed by stacking convolutional layers and connecting fully connected layers, and uses the relu activation function in a unified way. Common models include VGG16/VGG19
ResNet^41^	ResNet introduced residual structure to alleviate the problem of gradient disappearance/explosion, commonly used structure Resnet 34/50/101 and so on
WideResNet^42^	On the basis of ResNet, the network width is increased to further improve the training speed of the network. Common structures include WRN16-2/WRN40-2, etc
MobileNet^17^	MobileNet uses deep separable convolution instead of normal convolution to make the model more lightweight. Common structures include MobileNetv2/v3
ShuffleNet^38,39^	ShuffleNet uses point-by-point grouping convolution and channel rearrangement to reduce the model size. The common structure is ShuffleNetv1/v2

In order to ensure the fairness of experimental results, the results of existing methods are either reported in their articles^18–20^ or obtained using the code provided by them with exactly the same Settings. All results of CIFAR100 are the average of 5 trials, while the results of ImageNet are the average of 3 trials.

### Exploration of AFB

In this section, we first explore the effectiveness of transferring AFB, and then propose that learning continuous reasoning knowledge has greater benefits for students.

#### AFB contains more complete inference information than CAM and output features

Since the reasoning process is closely related to the reasoning outcome, a single transfer of the characteristics associated with the reasoning outcome from the teacher can cause students to imitate the incorrect reasoning process. The AFB contains complete inference information and theoretically gives the student a benefit, a gain that can be directly observed through the student's performance on the classification task.

We transferred different information from ResNet32 × 4 to ResNet8 × 4, including (1) only output features, (2) only CAM, and (3) AFB, and observed the performance on CIFAR-100. To align the teacher and student network, we divide the reasoning process into three stages based on ResNet's layer grouping. As shown in Table 2, transferring CAM or output features alone can also achieve good classification accuracy, but transferring AFB can further improve performance, suggesting that AFB carries more complete inference knowledge than CAM or output features, and this knowledge directly leads to student performance improvement. In addition, moving AFBs to different locations will bring different improvements to the performance of the subnetwork.

**Table 2:** Accuracy(%) of ResNet8 × 4 trained using different knowledge on cifar-100. The transferred knowledge are produced by ResNet32 × 4.

Knowledge	Feature	CAMs	AFB
Acc	75.34	73.89	77.14

#### Transferring continuous AFB is more beneficial to students

The forward propagation of data in the network has time continuity. The process from the input picture to the output prediction logic can be divided into different stages, and the reasoning results of the previous stage are used as the reasoning inputs of the later stage. Therefore, the reasoning information (including the reasoning process and reasoning results) of adjacent stages are also closely related. We have experimentally demonstrated that delivering continuous and complete AFB to students leads to more performance improvements.

Similarly, we use ResNet32 × 4 as the AFB producer and ResNet8 × 4 to learn the different stages of AFB. We divided the experiment into three groups: (1) only the AFB generated by Layer1 was studied; (2) Learn the AFB generated by Layer1-2; (3) Learn the AFB generated by all three layers. Layer1-3 corresponds to Stage1-3 in Fig. 1, where N equals 3.As shown in Table 3, learning only part of the inference knowledge has a similar classification accuracy (higher than the baseline network), but learning the full AFB results in a substantial improvement in network performance.

**Table 3:** Accuracy(%) of ResNet8 × 4 trained on cifar100 using knowledge from different layers.

AFB Producer	Layer-1	Layer1-2	Layer1-3
Acc	75.47	75.68	77.14

### Evaluation of AFT-KD

In this section, we compare AFT-KD with several popular knowledge distillation methods, including the feature-based KD method^18,22–25^, the logic-based KD method^20,21^, and the attention-graph-based KD method^19,30^.

#### Results on CIFAR-100

Similar to the work^19,20^, we carried out experiments on CIFAR-100 using the same teacher-student framework and different teacher-student framework respectively, and the Results were reported in Tables 4 and 5. Our approach implements new SOTA on both the same teacher-student architecture and some experiments with different teacher-student architectures. Among them, compared with the logic-based approach, our approach has achieved better results in all experiments with different architectures. However, when the student model is MobileNetV2, the performance of AFT-KD is slightly lower than^18,19^, which we speculate is due to the large difference between the teacher-student architecture and the simplistic alignment we used. In experiments with the same architecture, the accuracy of the student models trained by our method exceeded that of the teachers. Especially when the teacher model is ResNet32 × 4, the accuracy of AFT-KD reaches77.14%, which is 4.64% higher than the teacher model.

**Table 4:** Results on CIFAR-100. Teachers and students have different architectures.

Distillation manner	Teacher	ResNet32 × 4	WRN40-2	ResNet32 × 4	ResNet50	VGG13
	Acc	79.42	75.61	79.42	79.34	74.64
	Student	ShuffleNetV1	ShuffleNetV1	ShuffleNetV2	MobileNetV2	MobileNetV2
	Acc	70.5	70.5	71.82	64.6	64.6
Logits	KD^21^	74.07	74.83	74.45	67.35	67.37
	DKD^20^	76.45	76.7	77.07	70.35	69.71
Features	CRD^22^	75.11	76.05	75.65	69.11	69.73
	OFD^23^	75.98	75.85	76.82	69.04	69.48
	FitNet^24^	73.59	73.73	73.54	63.16	64.14
	RKD^25^	72.28	72.21	73.21	64.43	64.52
	ReviewKD^18^	77.45	77.14	77.78	69.89	**70.37**
Attention	AT^30^	71.73	73.32	72.73	58.58	59.4
	CAT-KD^19^	78.26	77.35	78.41	**71.36**	69.13
Ours	AFT-KD	**78.39**	**77.49**	**78.47**	70.48	69.22

Significant values are in [bold].

**Table 5:** Results on CIFAR-100. Teachers and students have the same architecture.

Distillation manner	Teacher	ResNet56	ResNet110	ResNet32 × 4	WRN-40–2	WRN-40–2	VGG13
	Acc	72.34	74.31	79.42	75.61	75.61	74.64
	Student	ResNet20	ResNet32	ResNet8 × 4	WRN-16–2	WRN-40–1	VGG8
	Acc	69.06	71.14	72.5	73.26	71.98	70.36
Logits	KD^21^	70.66	73.08	73.33	74.92	73.54	72.98
	DKD^20^	**71.97**	74.11	76.32	**76.24**	74.81	74.68
Features	CRD^22^	71.16	73.48	75.51	75.48	74.14	73.94
	OFD^23^	70.98	73.23	74.95	75.24	74.33	73.95
	FitNet^24^	69.21	71.06	73.5	73.58	72.24	71.02
	RKD^25^	69.61	71.82	71.9	73.35	72.22	71.48
	ReviewKD^18^	71.89	73.89	75.63	76.12	**75.09**	**74.84**
Attention	AT^30^	70.55	72.31	73.44	74.08	72.77	71.43
	CAT-KD^19^	71.62	73.62	76.91	75.6	74.82	74.65
Ours	AFT-KD	71.92	**74.23**	**77.14**	75.86	74.68	74.7

Significant values are in [bold].

#### Results on ImageNet

Tables 6 and 7 give the top-1 and top-5 accuracy of image classification on **ImageNet. Our method does not achieve the best performance due to the teacher's ability, but it is still better than most KD methods.

**Table 6:** Results on ImageNet. we set ResNet34 as the teacher and ResNet18 as the student.

Distillation manner			Features			Logits		Attention		Ours
Acc	Teacher	Student	OFD	CRD	ReviewKD	KD	DKD	AT	CAT-KD	AFT-KD
Top-1	73.31	69.75	70.81	71.17	71.61	70.66	**71.7**	70.69	71.26	71.47
Top-5	91.41	89.07	89.98	90.13	90.51	89.88	90.41	90.01	90.45	**90.61**

Significant values are in [bold].

**Table 7:** Results on ImageNet. we set ResNet50 as the teacher and MobileNet as the student.

Distillation manner			Features			Logits		Attention		Ours
Acc	Teacher	Student	OFD	CRD	ReviewKD	KD	DKD	AT	CAT-KD	AFT-KD
Top-1	76.16	68.87	71.25	71.37	**72.56**	68.58	72.05	69.56	72.24	72.44
Top-5	92.86	88.76	90.34	90.41	91.00	88.98	91.05	89.33	91.13	**91.35**

Significant values are in [bold].

**Figure 3:**
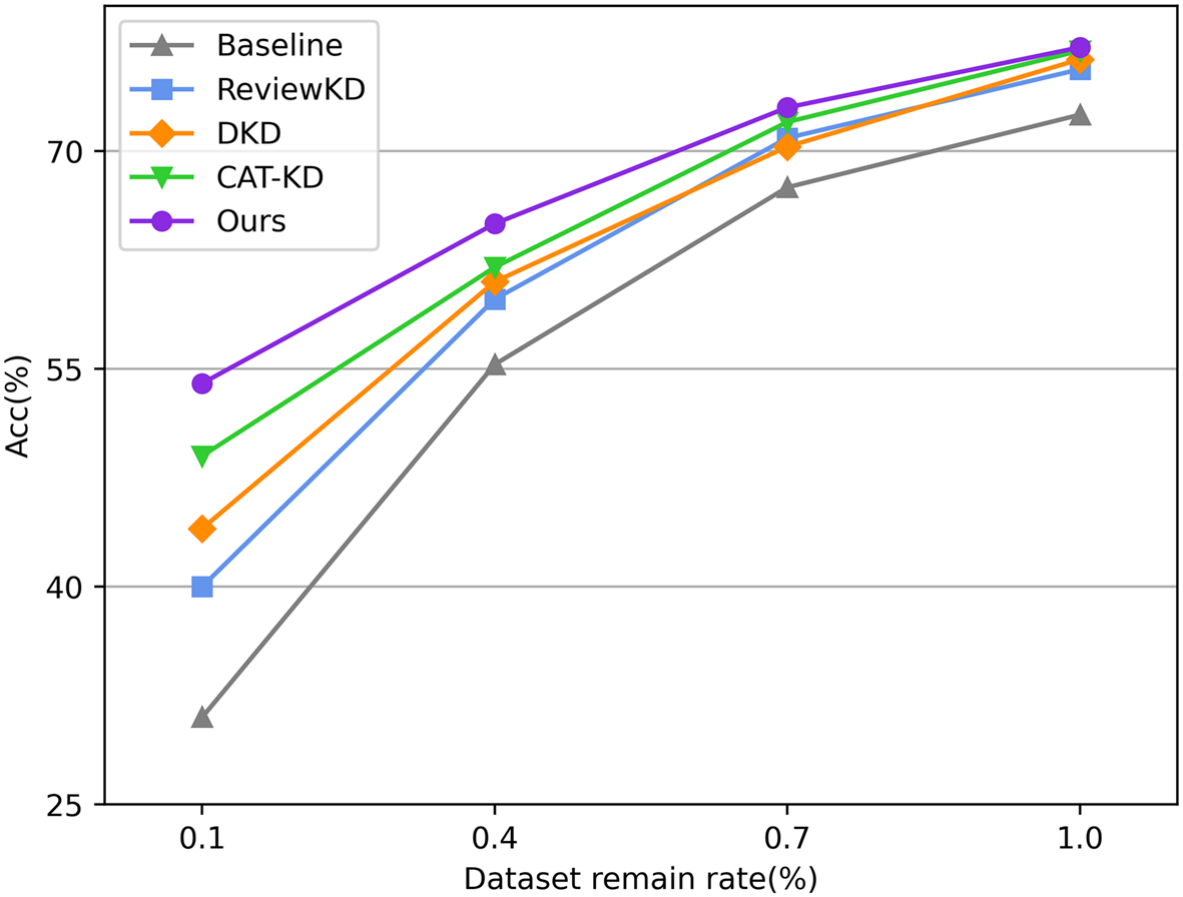
Accuracy(%) of student trained with several SOTA methods on the CIFAR-100. We set ResNet32 × 4 as the teacher and ResNet8 × 4 as the student, and the training set is reduced at various ratios.

Finally, we compare the performance of several SOTA methods on CIFAR-100, where the training set decays in different proportions, in line with the practice in^18^, and in doing so assess their dependence on the amount of training data. As shown in Fig. 3, AFT-KD is least affected by the amount of training data, demonstrating the excellent distillation efficiency of our method.

### Exploration of adaptive loss

**Figure 4:**
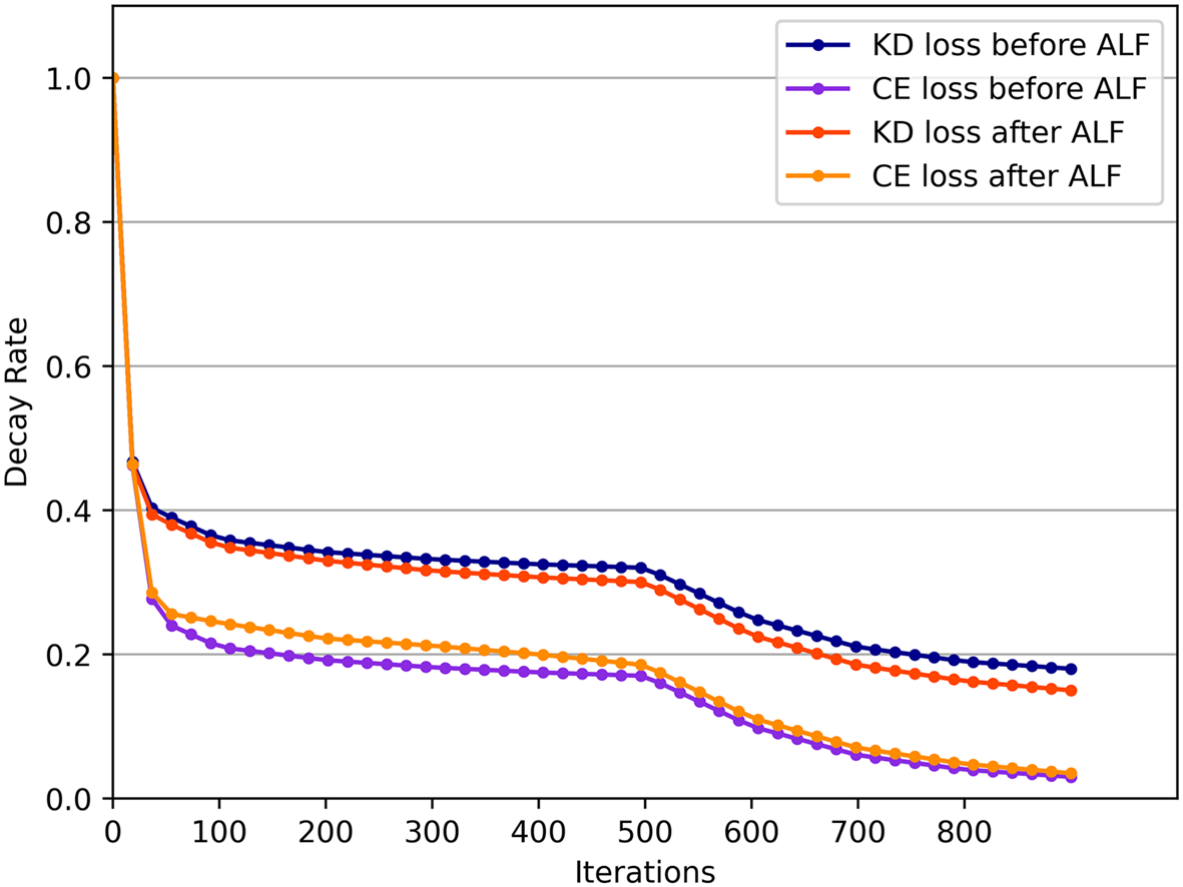
Comparison of optimized rate curves before and after using adaptive loss function.

From the analysis in section "Adaptive loss", we can see that the optimization rate imbalance of the loss function in multi-task learning will lead to the problem of decreased accuracy in the later training period. The adaptive loss function automatically adjusts the loss value according to the optimization rate of AFT loss and cross-entropy loss, which effectively alleviates the above problems. This improvement can be measured by the distance between the loss decay rate curves of multiple tasks, and Fig. 4 illustrates the comparison of the optimized rate curves before and after adopting the adaptive loss function. We sampled and computed the mean of the raw data for optimizing rates, resulting in a smoothed contrast curve. It can be observed that, after balancing the optimization rates of two losses using an adaptive loss function, the distance between the loss decay curves has decreased, and both losses are optimized in a more synchronized manner. This improvement has also effectively enhanced the model's classification performance.

## Conclusion

We analyze the existing KD methods and reclassify these methods into those based on the inference process and the inference result, which provides a new perspective for the study of knowledge distillation. Based on this, we propose AFT-KD with attention and feature transfer, which achieves competitive results on several commonly used benchmarks. Finally, in order to balance the loss optimization rate in AFT-KD, we propose an adaptive loss function based on loss decay rate to further improve the performance of AFT-KD. However, in experiments in which teachers and students adopt different architectures, the performance of AFT-KD is not the best among all methods, which we guess is caused by the large difference between teachers and students' architectures, and the alignment method we used is too simple. This limitation can be improved by designing specific alignment rules for network structures. In addition, compared with feature-based KD method, AFT-KD brings a certain improvement in operation cost, which is also something we need to continue to explore in future work.

## Data Availability

Contact Shuaiying Yu (6720210550@mail.jxust.edu.cn) for data and codes.

## References

[1] Liu Y, Wu H, Rezaee K (2022). Interaction-enhanced and time-aware graph convolutional network for successive point-of-interest recommendation in traveling enterprises. IEEE Trans. Industr. Inf..

[2] Grabek J, Cyganek B (2021). An impact of tensor-based data compression methods on deep neural network accuracy. Ann. Comput. Sci. Inf. Syst..

[3] Hameed MGA, Tahaei MS, Mosleh A (2022). Convolutional neural network compression through generalized Kronecker product decomposition. Proc. AAAI Confer. Artif. Intell..

[4] Hua W, Zhou Y, De Sa CM (2019). Channel gating neural networks. Adv. Neural Inf. Process. Syst..

[5] Gusak, J., Kholiavchenko, M., Ponomarev, E., *et al*. Automated multi-stage compression of neural networks. In *Proceedings of the IEEE/CVF International Conference on Computer Vision Workshops* (2019).

[6] Phan A. H., Sobolev, K., Sozykin, K., *et al*. Stable low-rank tensor decomposition for compression of convolutional neural network. In *Computer Vision–ECCV 2020: 16th European Conference, Glasgow, UK, August 23–28, 2020, Proceedings, Part XXIX 16* 522–539 (Springer International Publishing, 2020).

[7] Lin M, Ji R, Wang Y, et al. Hrank: Filter pruning using high-rank feature map. In *Proceedings of the IEEE/CVF Conference on Computer Vision and Pattern Recognition*, 1529–1538 (2020).

[8] Hou, Z., Qin, M., Sun, F., *et al*. Chex: Channel exploration for CNN model compression. In *Proceedings of the IEEE/CVF Conference on Computer Vision and Pattern Recognition*, 12287–12298 (2022).

[9] Fang, G., Ma, X., Song, M., *et al*. Depgraph: Towards any structural pruning. arXiv preprint arXiv:2301.12900 (2023).

[10] Ren, A., Zhang, T., Ye, S., *et al*. Admm-nn: An algorithm-hardware co-design framework of dnns using alternating direction methods of multipliers. In *Proceedings of the 24th International Conference on Architectural Support for Programming Languages and Operating Systems*, 925–938 (2019).

[11] Luo JH, Zhang H, Zhou HY (2018). Thinet: Pruning cnn filters for a thinner net. IEEE Trans. Pattern Anal. Mach. Intell..

[12] Cai, Y., Yao, Z., Dong, Z., *et al*. Zeroq: A novel zero shot quantization framework. In *Proceedings of the IEEE/CVF Conference on Computer Vision and Pattern Recognition*, 13169–13178. 2020.

[13] Xu, S., Li, H., Zhuang, B., *et al*. Generative low-bitwidth data free quantization. In *Computer Vision–ECCV 2020: 16th European Conference, Glasgow, UK, August 23–28, 2020, Proceedings, Part XII 16*, 1–17 (Springer, 2020).

[14] Li, H., Wu, X., Lv, F., *et al*. Hard sample matters a lot in zero-shot quantization. *Proceedings of the IEEE/CVF Conference on Computer Vision and Pattern Recognition*, 24417–24426 (2023).

[15] Howard, A., Sandler, M., Chu, G., *et al*. Searching for mobilenetv3. In *Proceedings of the IEEE/CVF International Conference on Computer Vision*, 1314–1324 (2019).

[16] Howard, A. G., Zhu, M., Chen, B., *et al*. *Mobilenets: Efficient convolutional neural networks for mobile vision applications*. arXiv preprint arXiv:1704.04861 (2017).

[17] Sandler, M., Howard, A., Zhu, M., *et al*. Mobilenetv2: Inverted residuals and linear bottlenecks. *Proceedings of the IEEE Conference on Computer Vision and Pattern Recognition*, 4510–4520 (2018).

[18] Chen, P., Liu, S., Zhao, H., *et al*. Distilling knowledge via knowledge review. In *Proceedings of the IEEE/CVF Conference on Computer Vision and Pattern Recognition*, 5008–5017 (2021).

[19] Guo, Z., Yan, H., Li, H., *et al*. Class attention transfer based knowledge distillation. In *Proceedings of the IEEE/CVF Conference on Computer Vision and Pattern Recognition*, 11868–11877 (2023).

[20] Zhao, B., Cui, Q., Song, R., *et al*. Decoupled knowledge distillation. In *Proceedings of the IEEE/CVF Conference on computer vision and pattern recognition*, 11953–11962 (2022).

[21] Hinton, G., Vinyals, O., Dean, J. Distilling the Knowledge in a Neural Network. arXiv preprint arXiv:1503.02531 (2015).

[22] Tian, Y., Krishnan, D., Isola, P. Contrastive representation distillation. arXiv preprint arXiv:1910.10699 (2019).

[23] Heo, B., Kim, J., Yun, S., *et al*. A comprehensive overhaul of feature distillation. In *Proceedings of the IEEE/CVF International Conference on Computer Vision*, 1921–1930 (2019).

[24] Romero, A., Ballas, N., Kahou, S. E., *et al*. Fitnets: Hints for thin deep nets. arXiv preprint arXiv:1412.6550 (2014).

[25] Park, W., Kim, D., Lu, Y., *et al*. Relational knowledge distillation. In *Proceedings of the IEEE/CVF Conference on Computer Vision and Pattern recognition*, 3967–3976 (2019).

[26] Peng, B., Jin, X., Liu, J., *et al*. Correlation congruence for knowledge distillation. In *Proceedings of the IEEE/CVF International Conference on Computer Vision*, 5007–5016 (2019).

[27] Tung, F., & Mori, G. Similarity-preserving knowledge distillation. *Proceedings of the IEEE/CVF International Conference on Computer Vision*, 1365–1374 (2019).

[28] Ji, M., Shin, S., Hwang, S., *et al*. Refine myself by teaching myself: Feature refinement via self-knowledge distillation. *Proceedings of the IEEE/CVF conference on computer vision and pattern recognition*, 10664–10673 (2021).

[29] Chen, L., Wang, D., Gan, Z., *et al*. Wasserstein contrastive representation distillation. In *Proceedings of the IEEE/CVF Conference on Computer Vision and Pattern Recognition*, 16296–16305 (2021)

[30] Komodakis, N., & Zagoruyko, S. Paying More Attention to Attention: Improving the Performance of Convolutional Neural Networks via Attention Transfer. ICLR. 2017.

[31] Cho, J. H., & Hariharan, B. On the efficacy of knowledge distillation. In *Proceedings of the IEEE/CVF International Conference on Computer Vision*, 4794–4802 (2019).

[32] Furlanello, T., Lipton, Z., Tschannen, M., *et al*. Born again neural networks. *International Conference on Machine Learning. PMLR*, 1607–1616 (2018).

[33] Mirzadeh SI, Farajtabar M, Li A (2020). Improved knowledge distillation via teacher assistant. Proc. AAAI Conf. Artif. Intell..

[34] Wang, J., Chen, Y., Zheng, Z., *et al*. CrossKD: Cross-Head Knowledge Distillation for Dense Object Detection. arXiv preprint arXiv:2306.11369 (2023).

[35] Passalis N, Tefas A (2018). Probabilistic Knowledge Transfer for Deep Representation Learning..

[36] Krizhevsky A, Hinton G (2009). Learning multiple layers of features from tiny images. Handb. Syst. Autoimmune Dis..

[37] Deng, J., Dong, W., Socher, R., *et al*. ImageNet : A Large-Scale Hierarchical Image Database. *Proc. CVPR* (2009).

[38] Ma, N., Zhang, X., Zheng, H. T., *et al*. Shufflenet v2: Practical guidelines for efficient cnn architecture design. In *Proceedings of the European Conference on Computer Vision (ECCV)*, 116–131 (2018).

[39] Zhang, X., Zhou, X., Lin, M., *et al*. Shufflenet: An extremely efficient convolutional neural network for mobile devices. *Proceedings of the IEEE Conference on Computer Vision and Pattern Recognition*, 6848–6856 (2018).

[40] Simonyan, K., & Zisserman, A. Very Deep Convolutional Networks for Large-Scale Image Recognition. arXiv preprint arXiv:1409.1556 (2014).

[41] He, K., Zhang, X., Ren, S., *et al*. Deep residual learning for image recognition. In *Proceedings of the IEEE Conference on Computer Vision and Pattern Recognition*, 770–778 (2016).

[42] Zagoruyko, S., & Komodakis, N. *Wide residual networks*. arXiv preprint arXiv:1605.07146 (2016).

