# Author Correction: Attention and feature transfer based knowledge distillation

**DOI:** 10.1038/s41598-023-47361-9

**Published:** 2023-11-20

**Authors:** Guoliang Yang, Shuaiying Yu, Yangyang Sheng, Hao Yang

**Affiliations:** School of Electrical Engineering and Automation, Jiangxi University of Science and Technology, Ganzhou, 341000 Jiangxi China

Correction to: *Scientific Reports* https://doi.org/10.1038/s41598-023-43986-y, published online 26 October 2023

The original version of this Article contained an error in Affiliation 1.

Affiliation 1

“School of Jiangxi, University of Science and Technology, Ganzhou, 341000, Jiangxi, China”

now reads:

Affiliation 1

“School of Electrical Engineering and Automation, Jiangxi University of Science and Technology, Ganzhou, 341000, Jiangxi, China”

The original Article has been corrected.

